# A Lightweight Certificateless Authenticated Key Agreement Scheme Based on Chebyshev Polynomials for the Internet of Drones

**DOI:** 10.3390/s25144286

**Published:** 2025-07-09

**Authors:** Zhaobin Li, Zheng Ju, Hong Zhao, Zhanzhen Wei, Gongjian Lan

**Affiliations:** Department of Electronic and Communication Engineering, Beijing Electronic Science and Technology Institute, Beijing 100070, China; lzb@besti.edu.cn (Z.L.); zh@besti.edu.cn (H.Z.); wzz@besti.edu.cn (Z.W.); 20233815@mail.besti.edu.cn (G.L.)

**Keywords:** Internet of Drones (IoD), Chebyshev polynomial, cetificateless, lightweight, authentication

## Abstract

**Highlights:**

**What are the main findings?**

- We propose a novel and practical certificateless cryptographic scheme utilizing Chebyshev polynomials.
- The proposed scheme significantly reduces computational overhead compared to existing solutions. Performance evaluations and comparative analysis reveal a substantial decrease in computational costs, with our scheme requiring approximately 65% less computational effort.

**What is the implication of the main finding?**

- This work fills a critical research gap by establishing a practical certificateless cryptographic scheme based on Chebyshev polynomials. Beyond this novelty, it also promotes the broader application and exploration of Chebyshev polynomials within the domain of public key cryptography.
- The considerable reduction in computational overhead, particularly when compared to certificateless schemes based on elliptic curve cryptography, positions our proposed solution as a highly attractive option for resource-constrained environments (e.g., the IoD).

**Abstract:**

The Internet of Drones (IoD) overcomes the physical limitations of traditional ground networks with its dynamic topology and 3D spatial flexibility, playing a crucial role in various fields. However, eavesdropping and spoofing attacks in open channel environments threaten data confidentiality and integrity, posing significant challenges to IoD communication. Existing foundational schemes in IoD primarily rely on symmetric cryptography and digital certificates. Symmetric cryptography suffers from key management challenges and static characteristics, making it unsuitable for IoD’s dynamic scenarios. Meanwhile, elliptic curve-based public key cryptography is constrained by high computational complexity and certificate management costs, rendering it impractical for resource-limited IoD nodes. This paper leverages the low computational overhead of Chebyshev polynomials to address the limited computational capability of nodes, proposing a certificateless public key cryptography scheme. Through the semigroup property, it constructs a lightweight authentication and key agreement protocol with identity privacy protection, resolving the security and performance trade-off in dynamic IoD environments. Security analysis and performance tests demonstrate that the proposed scheme resists various attacks while reducing computational overhead by 65% compared to other schemes. This work not only offers a lightweight certificateless cryptographic solution for IoD systems but also advances the engineering application of Chebyshev polynomials in asymmetric cryptography.

## 1. Introduction

After years of development, the unmanned aerial vehicle (UAV) has evolved from simple remote-controlled devices into an intelligent platform that integrates navigation, perception, and communication capabilities. With the advancements in modern communication technologies, a single UAV can no longer fulfill the collaborative requirements in increasingly complex scenarios. Therefore, in various applications such as emergency disaster relief, remote area coverage, and the Internet of Things (IoT), UAVs have formed the IoD through dynamic self-organizing networks. This novel, networked, intelligent, air-based communication system has emerged as a prominent focus of industrial and academic research [1,2,3]. As a key component of the emerging 6G communication systems, IoD breaks through the physical limitations of traditional terrestrial base stations by relying on its fast response capability and three-dimensional spatial topology flexibility. By utilizing dynamic deployment and seamless connectivity, IoD greatly extends the coverage of existing networks [4]. Nevertheless, the openness of the wireless channel during IoD communication results in IoD nodes being highly susceptible to various attacks, such as eavesdropping and forgery, when transmitting and processing data [5]. The security issues and resource-constrained challenges faced by UAVs severely limit the deployment of IoD for large-scale applications [6].

Nowadays, to address the security challenges of IoD, symmetric cryptography is commonly employed to secure UAV nodes. However, symmetric cryptography relies on pre-shared keys, which form the basis of symmetric encryption systems. If these keys are intercepted during the initial negotiation phase while being transmitted through an open channel, subsequent communications will be completely exposed [7]. Moreover, symmetric keys cannot be bound to the unique identities of UAVs, and any attacker can replicate a legitimate key to create fake nodes, thereby disrupting the operation of UAV swarms. The static key management mechanism of symmetric cryptography also proves ill-suited for the dynamic topological environment inherent in the IoD.

In contrast, public key cryptography can effectively deal with the security problem of IoD by strongly binding the public key with the identity to achieve forward security through dynamic key management. Certificate-based public key cryptography relies on a third-party certificate authority (CA) to manage the full life cycle of digital certificates, but there are certificate management issues. Identity-based cryptography (IBC) effectively removes the complexity of certificate management by using the user’s identity directly as a public key carrier, but it still suffers from key escrow problems. By combining partial private keys with the user’s secret values, certificateless public key cryptography (CL-PKC) [8] solves the key escrow problem of IBC. It retains the core advantages of certificate-free authentication. This cryptography paradigm combines lightweight features with enhanced security and holds considerable potential for engineering applications in resource-constrained IoD environments.

Nevertheless, the current mainstream certificateless cryptography schemes are generally based on elliptic curve cryptography (ECC). Although ECC shows good applicability in traditional network environments, the high computational complexity and significant energy consumption in its cryptography operations make it difficult to meet the IoD nodes’ dual demands of real-time response and lightweight computation. To address this bottleneck, Chebyshev polynomials with lightweight computational properties show unique advantages and provide a more adaptable cryptographic solution for IoD environments. The current cryptographic research based on Chebyshev polynomials focuses on symmetric cryptographic regimes, which are widely used in fields such as image encryption algorithm design because of their chaotic properties and fast iteration advantage. Chebyshev polynomials in public key cryptographic cryptosystems are still underexplored. Existing literature shows that although some scholars have proposed authentication and key negotiation protocols based on Chebyshev polynomials [9,10], compared with their mature applications in symmetric cryptography, critical issues such as the exploration of algebraic characteristics and the construction of security models for Chebyshev polynomials in public key cryptosystems still present research gaps. Their potential advantages as nonlinear mathematical tools in public key cryptography urgently require breakthroughs through algorithmic innovation and application expansion.

To address these problems, we introduce a certificateless cryptography scheme based on Chebyshev polynomials and propose an authentication and key negotiation protocol for the IoD. This solution addresses the critical issues of secure authentication and efficient key negotiation in the dynamic topologies of IoD environments.

The proposed scheme achieves a marked improvement in computational efficiency. However, it presents three potential avenues for further enhancement: (1) Its communication efficiency remains comparable to the baseline schemes, indicating no simultaneous improvement in this aspect. (2) Although the pseudo-identity (PID) mechanism provides fundamental privacy safeguards, it does not achieve complete dynamic anonymity. (3) The scheme proposed lacks quantum resistance.

### Contribution

The main contributions of this paper are as follows:

(1) Compared with the traditional public key cryptography mechanism, the proposed certificateless public key cryptography scheme not only improves the deployment flexibility of UAV nodes (e.g., allowing for rapid network access without pre-set certificates) but also enhances the system security by reducing the risk of key escrow.
(2) To address the computational constraints of IoD nodes, we introduce Chebyshev polynomials with lower computational overhead as certificateless cryptographic operators and construct a lightweight key negotiation protocol using their semigroup property.
(3) The security and performance analysis shows that the proposed scheme achieves a balance between security and resource overhead while guaranteeing IoD low-latency communication and dynamic topology adaptation.

## 2. Related Work

The authentication and key negotiation protocols between IoD nodes (e.g., UAVs and ground stations) are crucial in securing IoD communications. In 2024, Pu et al. [11] proposed a lightweight anonymous authentication and key negotiation protocol employing symmetric cryptography. This protocol generates session keys by using hash functions, XOR operations, and physical unclonable functions (PUF) to avoid sensitive data leakage risks in IoD. Similarly, Wazid et al. [12] introduced a novel three-factor authentication and key negotiation scheme between users and accessed UAVs, incorporating dynamic credentials to improve system flexibility. However, these symmetric cryptography-based protocols are prone to the proliferation of the number of keys stored by the nodes when applied to large-scale UAV clusters. At the same time, the characteristics of IoD’s multiple hops and rapid topology changes may also exacerbate the complexity of key management. Consequently, some scholars have explored public key cryptography based on elliptic curves to solve IoD authentication and key negotiation problems [13,14,15]. However, computational efficiency and certificate management issues cannot effectively meet the requirements of high real-time IoD. Certificateless cryptography, widely applied in the IoT and the Internet of Vehicles (IoV) [16,17,18], solves the certificate management problem but is still being explored in IoD environments where lower computational resource consumption is essential. Recently, since the computational cost of Chebyshev polynomials is merely one-third of the scalar multiplication in elliptic curve public key cryptography [9,19], utilizing Chebyshev chaotic maps to construct certificateless public key cryptography schemes offers a promising technological direction for enhancing IoD’s security [20].

The cryptographic application of chaotic maps began with the pioneering chaotic cryptographic system proposed by Habutsu et al. [21]. Initial research concentrated on the chaotic properties of Chebyshev polynomials, particularly their use in image encryption. Studies [22,23] have designed image encryption algorithms based on pixel permutation and grayscale value confusion using the polynomial’s high sensitivity to initial conditions and dynamic traversability. With further research, Chebyshev polynomials have found broader applications, such as in quantum image processing [24] and machine learning [25]; significantly, the semigroup property of Chebyshev polynomials has been gradually applied to the expansion of symmetric cryptosystems. In recent years, these polynomials have been integrated into the design of lightweight authentication protocols, resulting in several innovative proposals [10,26,27]. These proposals construct shared keys through polynomial iterative operations, achieving efficient key negotiation and conditional privacy protection in resource-constrained settings like IoV [10,26] and IoD [27]. Notably, the scheme in literature [26] employs dynamic identity mapping to achieve anonymous authentication of vehicle nodes while preserving polynomial computational efficiency. The scheme in document [27] capitalizes on the efficiency of chaotic maps to develop a lightweight authentication protocol for IoD, demonstrating significant advantages in computational and communication resource efficiency.

Compared to the flourishing developments in the symmetric cryptography domain, public key cryptography based on the Chebyshev polynomial has progressed more slowly. Kocarev et al. [28] first proposed the application of real-domain Chebyshev polynomials to public key cryptosystems, whose security is based on the problem of the intractability of polynomial iterations. However, Bergamo et al. [29] found that the periodicity of the cosine function in the real domain leads to polynomial trajectory predictability, allowing attackers to decrypt ciphertext efficiently without the private keys. Kocarev et al. [30] and Ning et al. [31] introduced the theoretical framework of Chebyshev polynomials under finite fields to overcome this flaw. They proved that it can construct public key schemes with the same security as the ElGamal algorithm [28]. Subsequently, Zhang [32] extended the use of the semigroup property by expanding the polynomial definition domain. Chen et al. [33] suggested that improper selection of modulus can result in overly short periods, making the system vulnerable to brute-force attacks. Therefore, it is crucial to choose an appropriate modulus to ensure that Chebyshev polynomials generate sequences with sufficiently long periods to resist such attacks.

Chebyshev polynomials also have extended applications in public key cryptography. In the field of certificateless public key cryptography, Algehawi and Samsudin [34] proposed a CL-PKC scheme based on Chebyshev polynomials, which significantly reduces the computational complexity by not using the bilinear pair operation. However, Tan et al. [35] pointed out a severe security vulnerability in the scheme, where attackers could derive the master key using linear correlations in the public key updating mechanism. Additionally, the scheme needs to generate temporary key pairs for each communication pair, leading to lower efficiency than the standard CL-PKC scheme. In subsequent research, Shakiba [36] proposed a multiplicative coupled cryptosystem by fusing the first and second classes of Chebyshev polynomials, improving key space complexity to resist brute-force attacks effectively. Recently, Lee [37] proposed an Identity-based Encryption (IBE) scheme using extended Chebyshev mapping to achieve chosen ciphertext security in the standard model with significantly reduced computation time compared to traditional schemes using bilinear pairing. Additionally, Long [38] innovatively combined blockchain with Chebyshev polynomial authentication schemes to construct a secure architecture for IoT supporting dynamic group key management, while Abdelfatah [39] proposed a model without secure channels that can simultaneously meet lightweight and non-repudiation requirements through key mixing mechanisms.

In summary, the above research indicates that elliptic curve-based cryptographic operations prevalent in mainstream public key cryptography face high resource consumption bottlenecks. Its high computational complexity and excessive storage requirements further limit its applicability in resource-constrained nodes within IoD systems. Although the cryptographic efficiency advantages of Chebyshev polynomials, supported by semigroup properties, have been thoroughly demonstrated, the current certificateless cryptography based on Chebyshev mapping remains incomplete. Existing schemes do not fully demonstrate computational efficiency advantages, and their foundational algorithmic designs do not entirely adhere to the principles of certificateless cryptography. This paper aims to address these flaws by constructing a certificateless cryptographic scheme based on the Chebyshev polynomial that offers lightweight security authentication and key negotiation protocols for IoD systems.

## 3. Preliminaries

### 3.1. Chebyshev Polynomial

**Definition** **1.** *(Chebyshev polynomial). Let n and x be variables such that* $n\in Z^{*}$*and x∈[−1,+1]. The cosine representation of the n-th order Chebyshev polynomial is defined as $T_{n}(x)=\cos(n\cdot \arccos\;(x))$. When n≥2, the equivalent recursive iterative definition is:*

$$\left\{\begin{array}{cc} 1 & \left(n=0\right)\\ x & \left(n=1\right)\\ 2xT_{n-1}\left(x\right)-T_{n-2}\left(x\right) & \left(n\geq 2\right) \end{array}\right.$$

### 3.2. Property

**Definition** **2.** *(Semigroup property): let* $r,\;s\in Z^{*}$*, x∈[−1,+1], the semigroup property of Chebyshev polynomials is defined as follows:*

$$\begin{array}{cc} T_{r}\left(T_{s\left(x\right)}\right) & =\cos\left(rcos^{-1}\left(\cos\left(scos^{-1}(x)\right)\right)\right)\\ & =\cos\left(rscos^{-1}\left(x\right)\right)\\ & =T_{rs}(x) \end{array}$$

Chebyshev polynomials also satisfy the exchange property: $T_{r}\left(T_{s}\left(x\right)\right)=T_{s}\left(T_{r}\left(x\right)\right)$.

In 2008, Zhang [28] proved that the semigroup property holds on the interval (−∞,+∞), which leads to a recursive relational formula under the extended domain of definition:

$$T_{n}\left(x\right)\;mod\;p=2xT_{n-1}\left(x\right)-T_{n-2}\left(x\right)\;mod\;p,\;n>2$$

where x∈(−∞,+∞) and *p* is a large prime number.

The extended Chebyshev polynomials also satisfy the exchange property: $T_{r}\left(T_{s}\left(x\right)\right)\;mod\;p=T_{s}\left(T_{r}\left(x\right)\right)mod\;p$.

**Definition** **3**  ([40])**.** *Let p be a large prime number,* $x\in Z_{p}$ *and $m,\;n\in Z_{p}^{*}$* *, then*

$$2T_{m}\left(x\right)T_{n}\left(x\right)\;mod\;p=T_{m+n}\left(x\right)\;mod\;p+T_{\left|m-n\right|}\left(x\right)\;mod\;p$$

### 3.3. Mathematically Hard Problems

**Definition** **4.** *(Chebyshev Discrete Logarithm Problem, CDLP): Given x and y, it is infeasible to find n by any polynomial time bounded algorithm, such that*  $y=T_{n}\left(x\right)$.

**Definition** **5.** *(Chebyshev Diffie-Hellman Problem, CDHP): Given x,*  $T_{n}\left(x\right)$ *and $T_{m}\left(x\right)$* *, the value of $T_{m\cdot n}\left(x\right)$* *cannot be solved by using any polynomial time bounded algorithm.*

## 4. Our Proposal

### 4.1. System Model

**Figure 1:**
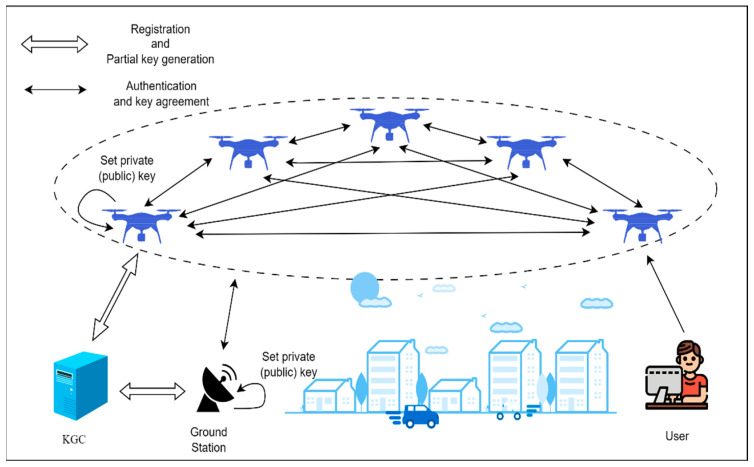
System model.

The system model for the Chebyshev polynomial-based certificateless authentication and key agreement protocol for the IoD proposed in this paper is illustrated in Figure 1.

The system comprises a key generation center (KGC) and IoD devices. In this structure, multiple UAVs must share task data in real-time, relying on efficient authentication key negotiation protocols to secure IoD communications. The process involves several stages: In the initialization phase, the KGC generates a master key pair and other system parameters. During the registration phase, entities such as UAVs and ground stations send registration requests and unique identifiers to the KGC. The KGC generates pseudonyms and public-private key pairs for each entity and sends these keys to the entities over a secure channel. In the complete key generation phase, entities such as UAVs aggregate the partial keys received from the KGC and combine them with their own secret values to generate complete key pairs. During the authentication and key negotiation phase, IoD nodes verify each other’s identities through signature verification and generate session keys through the key negotiation protocol to complete the authentication and key negotiation process.

### 4.2. Certificateless Authenticated Key Negotiation Based on Chebyshev Polynomials

**Figure 2:**
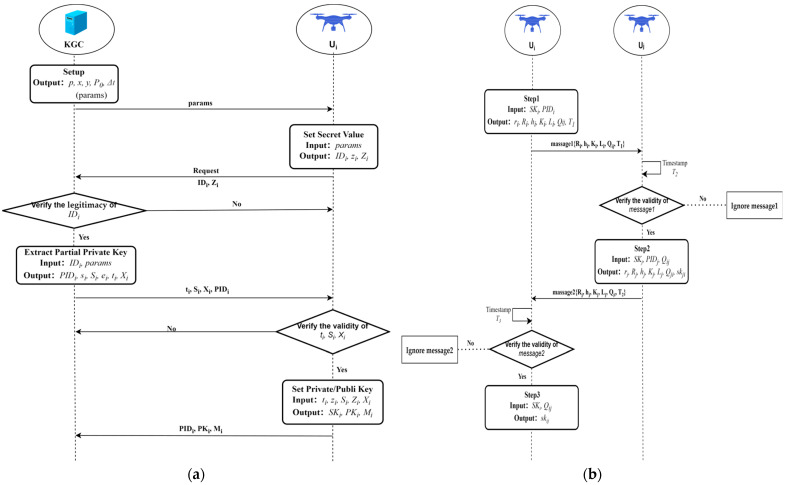
(**a**) System initialization and UAV registration; (**b**) Authenticated key agreement.

The detailed flow encompassing the initialization phase, registration phase, and authentication and key agreement phase of the proposed scheme is shown in Figure 2. Figure 2a illustrates the process of the scheme’s setup and registration, while Figure 2b presents the core steps of the scheme’s authenticated key agreement. The content depicted in Figure 2 will be elaborated upon in subsequent subsections of this chapter.

#### 4.2.1. Setup Phase

In this phase, KGC generates the master private key and public key based on Chebyshev polynomials according to the security requirements of the system, and generates other system parameters as follows:

(1) KGC picks a sufficiently large prime p and creates a unique identity $ID_{KGC}$.
(2) KGC chooses $x\in Z_{q}^{*}$ as the seed of a Chebyshev polynomial and a one-way secure hash function $H:\{0,1{\}}^{*}\to Z_{q}^{*}$.
(3) KGC chooses a random number $y\in Z_{q}^{*}$ as the system master private key and computes the corresponding system public key $P_{0}=T_{y}\left(x\right)modp$.
(4) KGC selects a request validity time Δt for the system.
(5) KGC publishes $\left\{p,H,x,P_{0},\Delta t\right\}$ to each user through a secure channel and secretly keeps its private key y.

#### 4.2.2. Authenticated Key Negotiation

In this phase, the UAV $U_{i}$ needs to interact with the KGC to complete the registration process, generating a self-complete public-private key pair as well as a pseudonym $PID_{i}$, which is used to realize identity anonymization and the subsequent key negotiation process as follows:

(1) $U_{i}$ selects a random number $z_{i}\in Z_{q}^{*}$, calculates $Z_{i}=T_{z_{i}}\left(x\right)modp$, and transmits a registration request containing information such as $\left\{ID_{i},Z_{i}\right\}$ to the KGC server through a secure channel.
(2) Upon receiving the registration request from $U_{i}$, the KGC verifies the legitimacy of $ID_{i}$ by checking it against a pre-established list of valid identities. If the $ID_{i}$ provided by $U_{i}$ is present in the identity list, $U_{i}$ is allowed to register; otherwise, the registration request from $U_{i}$ is denied.
(3) KGC uses the real identity $ID_{i}$ of UAV $U_{i}$ to compute $PID_{i}=H\left(ID_{i}\right)$ to generate the pseudonym $PID_{i}$.
(4) KGC picks a random number $s_{i}\in Z_{q}^{*}$, computes $S_{i}=T_{s_{i}}\left(x\right)modp$, $e_{i}=H\left(PID_{i},Z_{i},S_{i},P_{0}\right)$, $t_{i}=(s_{i}+ye_{i})modp$, and $X_{i}=T_{\left|s_{i}-e_{i}\cdot y\right|}\left(x\right)modp$, then returns the $\left\{t_{i},S_{i},X_{i},PID_{i}\right\}$ through the secure channel back to $U_{i}$.
(5) $U_{i}$ receives $\left\{t_{i},S_{i},X_{i},PID_{i}\right\}$, then calculates $PID_{i}=H\left(ID_{i}\right)$, $e_{i}=H\left(PID_{i},Z_{i},S_{i},P_{0}\right)$, and verifies $T_{t_{i}}\left(x\right)modp\overset{?}{=}2S_{i}T_{e_{i}}\left(P_{0}\right)modp-X_{i}$. If it holds, $U_{i}$ accept $\left\{t_{i},S_{i},X_{i},PID_{i}\right\}$; otherwise, ignore the message.
(6) $U_{i}$ obtains the complete public/private key pair $\left(SK_{i},PK_{i}\right)$, where $SK_{i}=\left(t_{i},z_{i}\right)$, $PK_{i}=\left(S_{i},Z_{i},X_{i}\right)$.
(7) $U_{i}$ precomputes $M_{i}=T_{\left|t_{i}-z_{i}\right|}\left(x\right)modp$, and sends $\left\{PK_{i},ID_{i},PID_{i},M_{i}\right\}$ through the secure channel to KGC.

#### 4.2.3. Authentication and Key Negotiation Phase

In this phase, assuming that $U_{i}$ and $U_{j}$ are two UAV nodes in the IoD and have generated their respective public/private key pairs after the registration phase, authentication and key negotiation can be accomplished through the following steps:

(1) $U_{i}$ chooses a random number $r_{i}\in Z_{q}^{*}$ and computes $R_{i}=T_{r_{i}}\left(x\right)modp$.
(2) $U_{i}$ takes the current timestamp $T_{1}$, calculates $h_{i}=H\left(PID_{i},R_{i},T_{1}\right)$, $K_{i}=\left(r_{i}+h_{i}\left(t_{i}+z_{i}\right)\right)modp$, $L_{i}=T_{\left|r_{i}-h_{i}\cdot \left(z_{i}+t_{i}\right)\right|}\left(x\right)modp$, $Q_{ij}=T_{r_{i}\left(t_{i}+z_{i}\right)}\left(x\right)modp$, and sends $\left\{PID_{i},R_{i},K_{i},L_{i},Q_{ij},T_{1}\right\}$ to $U_{j}$.
(3) $U_{j}$ receives the message $\left\{PID_{i},R_{i},K_{i},L_{i},Q_{ij},T_{1}\right\}$, obtains the current timestamp $T_{2}$, checks whether $\left|T_{2}-T_{1}\right|<\Delta t$ is valid, and retrieves the legitimacy of the $PID_{i}$ and obtains the $M_{i}$ via KGC. If any of the above conditions are not satisfied, $U_{j}$ ignores the message.
(4) $U_{j}$ calculates $h_{i}=H\left(PID_{i},R_{i},T_{1}\right)$, $e_{i}=H\left(PID_{i},Z_{i},S_{i},P_{0}\right)$ and verifies that $T_{K_{i}}\left(x\right)modp\overset{?}{=}\left(2R_{i}T_{h_{i}}\left(2\left(2S_{i}T_{e_{i}}\left(P_{0}\right)modp-X_{i}\right)Z_{i}modp-M_{i}\right)modp-L_{i}\right)modp$, if it is valid, then continue with the following process. Otherwise, the authentication is rejected.
(5) $U_{j}$ chooses the random number $r_{j}\in Z_{q}^{*}$ and computes the session key $sk_{ji}=T_{r_{j}\left(t_{j}+z_{j}\right)}\left(Q_{ji}\right)modp$.
(6) $U_{j}$ computes $R_{j}=T_{r_{j}}\left(x\right)modp$, $h_{j}=H\left(PID_{U_{j}},R_{j},T_{2}\right)$, $K_{j}=\left(r_{j}+h_{j}\left(t_{j}+z_{j}\right)\right)modp$, $L_{j}=T_{|r_{j}-h_{j}\cdot \left(z_{j}+t_{j}\right)}\left(x\right)modp$, $Q_{ji}=T_{r_{j}\left(t_{j}+z_{j}\right)}\left(x\right)modp$, and send $\left\{PID_{j},R_{j},K_{j},L_{j},Q_{ji},T_{2}\right\}$ to $U_{i}$.
(7) Receiving the message $\left\{PID_{j},R_{j},K_{j},L_{j},Q_{ji},T_{2}\right\}$, $U_{i}$ obtains the current timestamp $T_{3}$, checks whether $\left|T_{3}-T_{2}\right|<\Delta t$ is valid, and retrieves the legitimacy of $PID_{j}$ and obtains $M_{j}$ via KGC. If any of the above conditions are not satisfied, $U_{i}$ ignores the message.
(8) $U_{i}$ calculates $h_{j}=H\left(PID_{U_{j}},R_{j},T_{2}\right)$, $e_{i}=H\left(PID_{j},Z_{j},S_{j},P_{0}\right)$ and verifies that $T_{K_{j}}\left(x\right)modp\overset{?}{=}\left(2R_{j}T_{h_{j}}\left(2\left(2S_{j}T_{e_{j}}\left(P_{0}\right)modp-X_{j}\right)Z_{j}modp-M_{j}\right)modp-L_{j}\right)modp$. If it holds, $U_{i}$ computes the session key $sk_{ij}=T_{r_{i}\left(t_{i}+z_{i}\right)}\left(Q_{ji}\right)modp$.

After the above steps, the UAV nodes $U_{i}$ and $U_{j}$ will obtain the same session key, i.e., $sk_{ij}=sk_{ji}=T_{r_{i}r_{j}\left(t_{i}+z_{i}\right)\left(t_{j}+z_{j}\right)}\left(x\right)modp$.

#### 4.2.4. Correctness Analysis

In this section, we will provide a correctness analysis of the critical verification steps of the proposed cryptographic scheme. This analysis primarily relies on the two properties introduced in Section 3.2 for formal proof.

$$\begin{array}{c} T_{s_{i}+e_{i}\cdot y}\left(x\right)mod\;p=2\left(T_{s_{i}}\left(x\right)mod\;p\right)\left(T_{e_{i}}\left(T_{y}\left(x\right)mod\;p\right)mod\;p\right)-T_{\left|s_{i}-e_{i}\cdot y\right|}\left(x\right)mod\;p\\ =2S_{i}T_{e_{i}}\left(P_{0}\right)mod\;p-X_{i} \end{array}$$

$$\begin{array}{c} T_{K_{i}}\left(x\right)mod\;p=T_{r_{i}+h_{i}\left(t_{i}+z_{i}\right)}\left(x\right)mod\;p\\ \;=2T_{r_{i}}\left(x\right)mod\;p\cdot T_{h_{i}\left(t_{i}+z_{i}\right)}\left(x\right)mod\;p-T_{\left|r_{i}-h_{i}\left(z_{i}+t_{i}\right)\right|}\left(x\right)mod\;p\\ \;=2R_{i}T_{h_{i}}\left(T_{t_{i}+z_{i}}\left(x\right)mod\;p\right)mod\;p-L_{i}\\ \;=2R_{i}T_{h_{i}}\left(2T_{t_{i}}\left(x\right){mod\;p\cdot T}_{z_{i}}\left(x\right)mod\;p-T_{\left|t_{i}-z_{i}\right|}\left(x\right)mod\;p\right)mod\;p-L_{i}\\ \;=2R_{i}T_{h_{i}}\left(2\left(2S_{i}T_{e_{i}}\left(P_{0}\right)mod\;p-X_{i}\right)Z_{i}-M_{i}\right)mod\;p-L_{i} \end{array}$$

### 4.3. Informal Security Analysis

Here, the security of the proposed scheme is informally analyzed, which shows that the scheme is robust to well-known adversarial attacks.

#### 4.3.1. Device Capture Attack

In our scheme, even if a legitimate UAV device is physically captured and an adversary gains access to its public key $PK_{i}=\left(S_{i},Z_{i},X_{i}\right)$ and private key $SK_{i}=\left(t_{i},z_{i}\right)$ through techniques like power analysis, it remains computationally infeasible for the adversary to find $s_{i}$ from $Z_{q}^{*}$ such that $S_{i}=T_{s_{i}}\left(x\right)$ based on the CDLP assumption. Consequently, without knowing $s_{i}$, the adversary cannot derive the system master private key y from $t_{i}=s_{i}+ye_{i}$, preventing impersonation of the KGC.

#### 4.3.2. Forward Secrecy

Forward secrecy ensures that even if a user’s long-term key is compromised or leaked, the confidentiality of previous session keys is not endangered, thus protecting past communication. In our protocol, the session key $sk=T_{r_{i}r_{j}\left(t_{i}+z_{i}\right)\left(t_{j}+z_{j}\right)}\left(x\right)$ is jointly generated from random numbers and private keys. It varies with each generation due to differing random numbers, making it impossible for the adversary to derive the session key sk solely from the user’s private key. Therefore, the protocol achieves forward security.

#### 4.3.3. Man-in-the-Middle Attack

A man-in-the-middle attacker establishes a secret connection between two communicating parties, intercepting, altering, or forwarding their communications to steal sensitive information or disrupt communication integrity. In our protocol, UAVs $U_{i}$ and $U_{j}$ can only share a session key $sk_{ij}$ after mutual authentication, preventing the attacker from establishing a legitimate session with either $U_{i}$ or $U_{j}$. Thus, the protocol is secure against man-in-the-middle attacks.

#### 4.3.4. Replay Attack

In the replay attack, the adversary intercepts information transmitted during IoD drone communications and replays authentication messages in the current session to impersonate legitimate UAVs. In our protocol, message senders include a current timestamp in the authentication signature, allowing receivers to detect replayed messages by verifying the timestamp’s validity. Thus, the protocol is secure against replay attacks.

#### 4.3.5. Identity Privacy Protection

Our scheme uses pseudonyms derived from the real identities of UAVs through hash functions, $PID_{i}=H\left(ID_{i}\right)$, for communication between UAVs. The one-way property of hash functions ensures that sensitive information, such as the UAVs’ true identities, is not disclosed to other member UAVs, and adversaries find it impossible to extract real identities from transmitted messages. Therefore, our protocol provides identity privacy protection.

#### 4.3.6. Key Leakage Attack

Key leakage attack implies that an adversary attempts to obtain user keys by intercepting negotiation and authentication information during the key agreement process. In our protocol, even if the adversary intercepts $U_{i}$‘s authentication message $K_{i}=r_{i}+h_{i}\left(t_{i}+z_{i}\right)$ and $R_{i}=T_{r_{i}}\left(x\right)$, the probability of finding a legitimate key $\left(t_{i}+z_{i}\right)$ in $Z_{q}^{*}$ is negligible based on the CDLP assumption. Thus, the adversary cannot obtain $U_{i}$‘s key information or impersonate $U_{i}$ to communicate with $U_{j}$, making the protocol resistant to key leakage attacks.

#### 4.3.7. Eavesdropping Attack

Eavesdropping attack implies that an adversary intercepts the authentication information of both communicating parties during the key negotiation process and attempts to derive the session key. If an adversary intercepts both $U_{i}$‘s authentication message $Q_{ij}=T_{t_{i}+z_{i}}\left(T_{r_{i}}\left(x\right)\right)$ and $U_{j}$‘s message $Q_{ji}=T_{t_{j}+z_{j}}\left(T_{r_{j}}\left(x\right)\right)$, the Chebyshev polynomial-based CDHP assumption makes the probability of obtaining the session key $sk_{ij}=sk_{ji}=T_{r_{i}r_{j}\left(t_{i}+z_{i}\right)\left(t_{j}+z_{j}\right)}\left(x\right)$ negligible. Hence, the protocol can prevent eavesdropping attacks.

#### 4.3.8. Impersonation Attack

An impersonation attack implies that an attacker uses captured messages to impersonate a legitimate participant and communicate with the other party. In this scheme, the attacker wishing to impersonate $U_{i}$ needs to generate authentication messages $\left\{PID_{i},R_{i},K_{i},L_{i},M_{i},T_{1}\right\}$ from accessible data. Without knowing r and the private key $SK_{i}$, the attacker cannot forge $R_{i},K_{i},L_{i}$. Thus, the protocol resists impersonation attacks.

#### 4.3.9. Temporary Key Leakage Attack

Resistance to temporary key leakage ensures that even if temporary keys are leaked, the long-term private keys and session keys of UAV devices remain secure. In this protocol, temporary keys $r_{i},r_{j}$ are randomly generated and independent of long-term private keys $SK_{i}$, $SK_{j}$ with the session key $sk=T_{r_{i}r_{j}\left(t_{i}+z_{i}\right)\left(t_{j}+z_{j}\right)}\left(x\right)$ derived from random numbers and private keys. Consequently, even if temporary keys are leaked, adversaries cannot derive session keys or long-term private keys SK.

#### 4.3.10. Insider Privilege Attack

In our certificateless scheme, the private key is composed of $t_{i}$ and $z_{i}$, where $t_{i}=s_{i}+ye_{i}$ is generated by the KGC selecting random parameters $s_{i}$ and signing with the master private key y, and $z_{i}$ is a user-selected random number. During registration, UAVs send registration information $\left\{ID_{i},\;\;Z_{i}\right\}$ to the KGC, and based on the CDLP assumption, the KGC cannot derive $z_{i}$ from $Z_{i}$. Even if the KGC maliciously leaks $t_{i}$, since $z_{i}$ is retained by the user, adversaries cannot obtain the complete private key $SK_{i}$, thus preventing insider privilege attacks.

## 5. Performance Analysis

### 5.1. Scheme Computational Cost

In this section, we compared the computational costs of our scheme with four other related schemes. In our experiments, we used the Miracl and GMP libraries to simulate these schemes on a Windows system, utilizing an AMD Ryzen 7 5800H 3.20-GHz processor (Santa Clara, CA, USA) with 16 GB RAM. Our simulations employed the secp160r1 elliptic curve parameters and SHA256 as the hash function. Additionally, the bit length for modular operations was set at 256 bits, and the bit length for Chebyshev polynomial operations was 160 bits.

**Figure 3:**
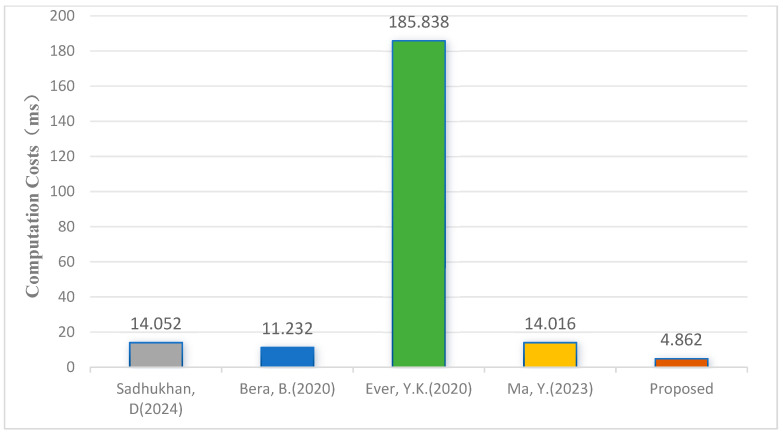
Computation costs [13,14,15,16].

Computational efficiency refers to the time consumed by different cryptographic operations during identity authentication and key agreement protocols. The symbols $T_{H}$, $T_{bp}$, $T_{ecm}$, $T_{eca}$, and $T_{c}$ represent the time for executing one-way hash functions, bilinear pairing operations, elliptic curve point multiplication, elliptic curve point addition, and Chebyshev polynomial operations, respectively. The simulation results presented in Table 1 show that $T_{H}$ is approximately 0.002 ms, $T_{bp}$ is about 30.034 ms, $T_{ecm}$ is about 1.400 ms, $T_{eca}$ is about 0.006 ms, and $T_{c}$ is about 0.485 ms. Our protocol implements bidirectional authentication key agreement, requiring 14 Chebyshev polynomials and 6 hash function operations during the authentication and key agreement phase. Consequently, the computational cost of our proposed protocol is $\left(14\times 0.144\right)+\left(6\times 0.004\right)=2.04\;ms$. Table 2 and Figure 3 compare the computational costs of our protocol with other schemes from different dimensions.

The results show that our protocol offers higher computational efficiency. The primary reason is that our approach employs Chebyshev polynomials, which are more lightweight than elliptic curve point multiplication, as cryptographic operators. Compared to references [13,14,15], our protocol uses a certificateless structure, avoiding certificate management and key escrow issues and providing a more efficient solution for authentication and key agreement in resource-constrained IoD devices.

### 5.2. Scheme Communication Cost

**Figure 4:**
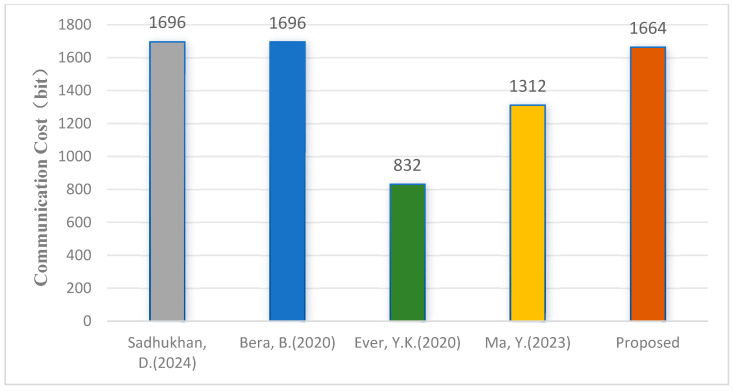
Communication costs [13,14,15,16].

When considering communication efficiency, only communication messages related to authentication are counted. Assume that the size for identity ID, hash values/random numbers, timestamps, ECC points, Chebyshev polynomial output values, and encryption/decryption parameters are 160 bits, 160 bits, 32 bits, 320 bits, and 160 bits, respectively. The key agreement process in our proposed protocol involves two messages, resulting in a total communication cost of 10×160+2×32=1664 bit. The comparison of total communication costs with other schemes is shown in Figure 4. The results indicate that the communication cost of our proposed scheme is slightly higher than schemes [15,16] but lower than schemes [13,14].

## 6. Conclusions

This paper addresses the dual challenges of dynamic topology and resource constraints in the IoD environment by proposing a certificateless authentication and key agreement scheme based on Chebyshev polynomials. Theoretical analysis and simulation results demonstrate that this scheme introduces the lightweight characteristics of Chebyshev chaotic mapping into the certificateless public key cryptographic architecture, resolving the certificate management issues in existing IoD public key authentication solutions. Compared to traditional elliptic curve cryptographic schemes, there is a significant improvement in computational efficiency.

Future research will focus on three directions: (1) exploring the collaborative security mechanisms of Chebyshev polynomials with emerging technologies such as blockchain and federated learning to construct a cross-domain authentication system for the IoD environment, (2) incorporating a periodic identity update mechanism to strengthen privacy protection, and (3) exploring hybrid post-quantum cryptographic extensions to fortify quantum resistance.

## Figures and Tables

**Table 1:** Execution time of cryptographic elements.

Notation	Description	Cost
$T_{H}$	Time of one-way hash operation	≈0.002 ms
$T_{H}$	Time of a bilinear pairing	≈30.034 ms
$T_{ecm}$	Time of an EC point multiplication	≈1.400 ms
$T_{eca}$	Time of an EC point addition	≈0.006 ms
$T_{c}$	Time of a Chebyshev polynomial computation	≈0.485 ms

**Table 2:** Computation overhead comparison with other related protocols.

Schemes	Operations	Total Cost
Scheme [13]	$10\;T_{ecm}+14\;T_{H}+4\;T_{eca}$	14.052 ms
Scheme [14]	$8{\;T}_{ecm}+10\;T_{H}+2\;T_{eca}$	11.232 ms
Scheme [15]	$4{\;T}_{ecm}+17{\;T}_{H}+6\;T_{bp}$	185.838 ms
Scheme [16]	$10{\;T}_{ecm}+5\;T_{H}+T_{eca}$	14.016 ms
Proposed	$14\;T_{c}+6\;T_{H}$	4.862 ms

## Data Availability

Data are contained within the article.

## Abbreviations

The following abbreviations are used in this manuscript:

IoD	Internet of Drones
UAV	Unmanned Aerial Vehicle
IoT	Internet of Things
CA	Certificate Authority
IBC	Identity-based Cryptography
CL-PKC	Certificateless Public Key Cryptography
ECC	Elliptic Curve Cryptography
PUF	Physical Unclonable Functions
IoV	Internet of Vehicles
IBE	Identity-based Encryption
CDLP	Chebyshev Discrete Logarithm Problem
CDHP	Chebyshev Diffie-Hellman Problem
KGC	Key Generation Center

Definition of symbols for the scheme of this paper:

**Notion**	**Meaning**
$ID_{s}$	Identifier of $U_{s}$
T	Timestamp
$PID_{s}$	Pseudonym of $U_{s}$
$SK_{s}/PK_{s}$	Private/Public key of $U_{s}$
$y/P_{0}$	Private/Public key of KGC
H(·)	cryptographic hash function
p,q	large prime number
x	Seed of Chebyshev polynomials
$T_{n}\left(x\right)$	Chebyshev polynomials
$sk_{sl}$	Session key between $U_{s}$ and $U_{l}$
